# Lyn regulates epithelial–mesenchymal transition in CS-exposed model through Smad2/3 signaling

**DOI:** 10.1186/s12931-019-1166-z

**Published:** 2019-09-02

**Authors:** Xiaobo Liang, Xiang He, Yin Li, Junyi Wang, Dehong Wu, Xiefang Yuan, Xiaoyun Wang, Guoping Li

**Affiliations:** 1 Inflammation & Allergic Diseases Research Unit, Affiliated Hospital of Southwest Medical University, Luzhou, 646000 China; 2 First Department of Respiratory Disease, Affiliated Hospital of Southwest Medical University, Luzhou, 646000 China; 3 Laboratory of Allergy and Inflammation of Allergy Department, Chengdu Institute of Respiratory Health, the Third People’s Hospital of Chengdu, Affiliated Hospital of Southwest Jiaotong University, Chengdu, 610031 China; 4 Department of Respiratory Disease, the Third People’s Hospital of Chengdu, Affiliated Hospital of Southwest Jiaotong University, Chengdu, 610031 China; 5 Department of Thoracic Surgery, Zhongshan Hospital, Affiliated Hospital of Fudan University, Shanghai, 200032 China

**Keywords:** Lyn, EMT, COPD, Smad2/3

## Abstract

**Background:**

Chronic obstructive pulmonary disease (COPD) is characterized by airflow limitation that is progressive and not fully reversible. Cigarette smoking is one of the most commonly and important risk factors for COPD, which contributes to airway remodeling, the outstanding pathological changes in COPD. One potential mechanism which might be important for airway remodeling is the process called epithelial–mesenchymal transition (EMT). However, the underlying molecular mechanisms of EMT in CS-induced COPD are still poorly understood.

**Methods:**

Two Gene Expression Omnibus (GEO) datasets (GSE108134 and GSE5058) were combined to identify the key genes involved in COPD. Then, single-gene analysis of Lyn was performed. Lyn expression was confirmed in patients with COPD. 16HBE cells were treated with cigarette smoking extracts (CSE). Wild type (WT) C57BL/6 J mice and Lyn+/+ transgenic mice were exposed to CSE to establish CS-exposed model. Pathological changes were observed by hematoxylin-eosin staining. The expression levels of EMT markers were examined by using western blot and immunofluorescence. The expression and phosphorylation levels of Lyn and Smad2/3 were detected as well.

**Results:**

The gain of mesenchymal markers vimentin and α-SMA with a concomitant loss of E-cadherin was observed in both in vivo and in vitro studies. Meanwhile, cigarette smoking extracts (CSE) induced EMT in 16HBE cells in a time- and dose- dependent manner. Furthermore, by analyzing GEO datasets and using molecular methods, we explored a kinase, Lyn, its expression correlated with the expression of E-cadherin, vimentin and α-SMA in CS-exposed model. Moreover, we found that EMT induced by CSE was regulated by activated Lyn through phosphorylation of Smad2/3.

**Conclusions:**

In summary, we found that Lyn regulates epithelial–mesenchymal transition in CS-exposed model through Smad2/3 signaling. As a kinase, Lyn is “druggable”, and might provide a therapeutic opportunity for targeting EMT. Therefore, our research might provide a new method to treat COPD by targeting Lyn kinase specifically.

## Background

Chronic obstructive pulmonary disease (COPD), which is characterized by continuous limitation of airflow, is a chronic inflammatory pulmonary disease. According to the data of World Health Organization, COPD will be the third leading cause of death all over the world by the year 2030 [1]. Cigarette smoking is one of the most commonly and important risk factors for COPD. Although a few of individuals with COPD had never smoked, personal exposure to tobacco smoke like cigarette smoking is one of the best known risk factor for COPD [2]. It was found that the prevalence rate of COPD in smokers is 3 to 5-times higher than that in nonsmokers [3]. However, the underlying molecular mechanisms of cigarette smoking in COPD development are still poorly understood.

Remodeling of the airways is one of the outstanding pathophysiological changes in COPD, which consists of detrimental changes in structural tissues and cells including epithelial metaplasia, smooth muscle hyperplasia, goblet cell hypertrophy, and airway wall thickening [4, 5]. Airway remodeling largely leads to airway obstruction and causes the airflow limitation seen in patients with COPD. However, the mechanism of airway remodeling is not fully elucidated. One potential mechanism which might be important for this pathology is the process of epithelial–mesenchymal transition (EMT) [6, 7]. EMT is a biological process in which epithelial cells lose their abilities of cell–cell adhesion and polarity with rearrangement of the cytoskeleton, and the cells acquire the abilities of producing ECM components, invasion and migration [8, 9]. Biomarkers of epithelial cells like E-cadherin and ZO-1 are down-regulated during EMT, whereas mesenchymal markers, VIM (Vimentin) and α-SMA (alpha-smooth muscle actin), are up-regulated [10, 11]. Although the mechanism of EMT in COPD has not been fully illustrated, Smad2/3 signaling is believed to be involved in EMT. Smad2 and Smad3 belong to Smad-transcription factors, which are activated by TGFβ receptors. Phospho-activated Smad2/3 form a complex with Smad4, then the complex translocates to the nucleus. In the nucleus, this Smad complex binds to promoter regions of target genes and regulates expression of numerous target genes [12–14]. Recent studies also suggest that Smad2 and/or Smad3 are associated with EMT-related cancer models [15–17].

Lyn is a Src-family kinase, a family of non-receptor tyrosine kinases, which plays vital roles in signal regulation and transduction. Lyn has been implicated in regulation of cellular events, such as survival, proliferation and apoptosis depending on cell types and stimulus [18]. Studies have shown that Lyn plays a crucial role in Kaposi’s Sarcoma, autoimmune disease and hepatic cirrhosis as well as allergic bronchial asthma [19, 20]. In addition, Lyn is associated with metastasis and may promote EMT in tumor progression [21]. In this study, we explored the underlying mechanism of how Lyn regulates EMT in CS-exposed model. Lyn was activated in CS-exposed model and promoted EMT both in vitro and in vivo studies. Furthermore, the phosphorylation of Smad2/3 was up-regulated by activated Lyn. Our results suggest that Lyn is the activator of Smad2/3, the major transcription factors involved in EMT. Therefore, specific targeting of Lyn might be an effective therapeutic approach to treat COPD by inhibition of EMT.

## Materials and methods

### Reagents

Anti-Lyn(Santa Cruz, CA, sc-15), anti-Vimentin (Santa Cruz,sc-6260), anti-Smad2/3(Santa Cruz, CA, sc-8332), anti-p-Smad2/3(Santa Cruz, CA, sc-11,769), and anti-β-actin(Santa Cruz, CA, sc-130,656) were purchased from Santa Cruz Biotechnology. Anti-E-cadherin (Abcam,ab11512), and anti-α-SMA (Abcom,ab5694) were purchased from Abcam Biotechnology. Anti-p-Lyn (CST, #2731) was purchased from Cell Signaling Technology. Nonsilencing siRNA control, Lyn-specific siRNA were purchased from Santa Cruz Biotechnology (Santa Cruz, CA). Recombinant lentivirus containing Lyn or a GFP-only virus control was obtained from Cyagen (Shanghai, China).

### Animals and cell line

The construction of the Lyn^+/+^ transgenic mice with inbred C57BL/6 J genomic background has been previously described [22]. Genotyping was confirmed by western blotting. Wild type (WT) C57BL/6 J mice at 8 to 10 weeks of age were obtained from Tengxin Biotechnology Co., Ltd. (Chongqing, China). Lyn^+/+^ TG mice and WT mice were maintained in a specific pathogen-free facility and routinely monitored. All animal experiments were performed and approved in accordance with the guidelines of the Institutional Animal Care and Use Committee in Southwest Medical University. The 16HBE cell line was obtained from Wang [22].

### Bioinformatics and computational analysis

The published microarray datasets, GSE108134 and GSE5058, were obtained from public database NCBI. Airway epithelial cells from COPD-smoker or Non-smoker were obtained by bronchoscopy and brushing in both GEO datasets. The sample numbers of Non-smoker group are 39, COPD-smoker group are 69. The matrix data of GSE108134 and GSE5058 were merged and normalized by sva package with R software. Gene set enrichment analysis was performed with GESA 3.0 according to previous publications [23, 24].

### Establishment of murine CS-exposed model

To establish a murine CS-exposed model, 6 to10-weeks old Lyn^+/+^ transgenic mice and WT mice were treated with CSE combined with lipopolysaccharide (LPS), 5 mice in each group were used [25, 26]. The mice were treated with CSE for 5 days per week for a total of 6 weeks, 10 μg LPS was given twice a week for 6 weeks. Control mice were given PBS alone.

### Histology

Lung tissues were fixed in 10% neutral-buffered formalin for 24 h and then embedded in paraffin. The sections (5 μm) of the lung specimens were stained with standard hematoxylin-eosin staining (H&E) methods to assess histology. Histological review was performed in a blinded fashion by two independent pulmonary observers.

### Cell culture, viral infection and transfection

Human Lyn cDNA was amplified and cloned into the pLV.ExBi.P/Puro-EF1α-IRES-eGFP/ pLV.Des3d.P/Puro vector. The vector expressing Lyn was constructed and transfected into human embryo kidney cells 293 T cells to create infectious lentiviral vector [22]. Human airway epithelial cells 16HBE were obtained from American Type Culture Collection (ATCC; Manassas, USA). 16HBE cells were maintained in Dulbecco’s modified Eagle’s medium (DMEM) with 10% fetal bovine serum at 37 °C in with 5% CO2. 16HBE cells were grown to 70% confluence in DMEM culture media containing 10% fetal bovine serum. 16HBE cells were transfected with recombinant lentivirus containing Lyn or a GFP-only virus control according to the manufacturer’s instructions. 16HBE cells were transfected with lentivirus at a multiplicity of infection (MOI) of 5–10. The supernatant was exchanged for fresh medium after 12 h-inoculation. For Lyn interfering RNA (siRNA), cells were transfected with 20uM small interfering RNA using Lipofectamine 2000 according to the manufacturer’s instructions. The media were changed after 6 h-transfection. Cigarette smoke extracts (CSE) were prepared as previously outlined [27].The cells were treated with 0% CSE, 2.5% CSE and 5% CSE for 72 h, and 5% CSE at 6 h, 12 h, 24 h, 48 h, 72 h.

### Immunofluorescence

Frozen lung tissues sections (5 μm) and cells were fixed with ice-cold methanol and permeabilized in PBS containing 0.25% Triton X-100 for 10 min at room temperature. Nonspecific binding was blocked for 1 h with 1% BSA (Sigma-Aldrich, B2064) in PBS containing 0.05% Tween 20. Specimens were then incubated with antibodies against E-cadherin, vimentin and α-SMA. FITC-conjugated or TRITC-conjugated secondary Abs were used to probe the primary antibodies. After the specimens were washed, nuclei were stained with 4′-6-diamidino-2-phenylindole dihydrochloride (DAPI, Invitrogen). The specimens were analyzed using an SP5 Leica confocal microscope with Leica Application Suite Software (Version number: 14.0.0162, Leica, German). Isotype control was replaced the Abs as the negative control.

### Western blotting

Lung tissues or cells were homogenized in liquid nitrogen and re-suspended in radioimmunoprecipitation assay (RIPA) buffer with optional protease inhibitor cocktail (Roche Applied Science, Indianapolis, USA). The protein concentrations were measured using a Nanodrop Instrument (Epoch, BioTek). Protein samples were separated in 8–10% SDS-PAGE at 100 V for 90 min and transferred onto a microporous polyvinylidene difluoride (PVDF) membrane with 250 mA current for 1 h using a wet transfer method. The PVDF membranes were incubated with antibodies against E-cadherin, vimentin, α-SMA, Smad2/3, p-Smad2/3, β-actin, Lyn, and p-Lyn. Horseradish peroxidase (HRP)-conjugated secondary antibodies were used to bind corresponding primary antibodies and visualized via an enhanced chemiluminescence kit. The band intensity was evaluated against the loading control to derive a ratio value using Quantity One software.

### Statistical analysis

All statistical analyses were performed using the SPSS 17.0 software (Chicago, IL, USA). Graphs were made in Graphpad Prism 7 (GraphPad Software Inc., LaJolla, CA). Quantitative data are presented as the mean ± s.d. Multiple group comparisons were analyzed using a one-way ANOVA. Two group comparisons were analyzed by using the Tukey-Kramer post-test or Dunnett’s T3. Statistical significance was defined as a *P* value < 0.05.

## Results

### The expression levels of EMT makers correlate with Lyn in COPD-smoker patients

**Fig. 1:**
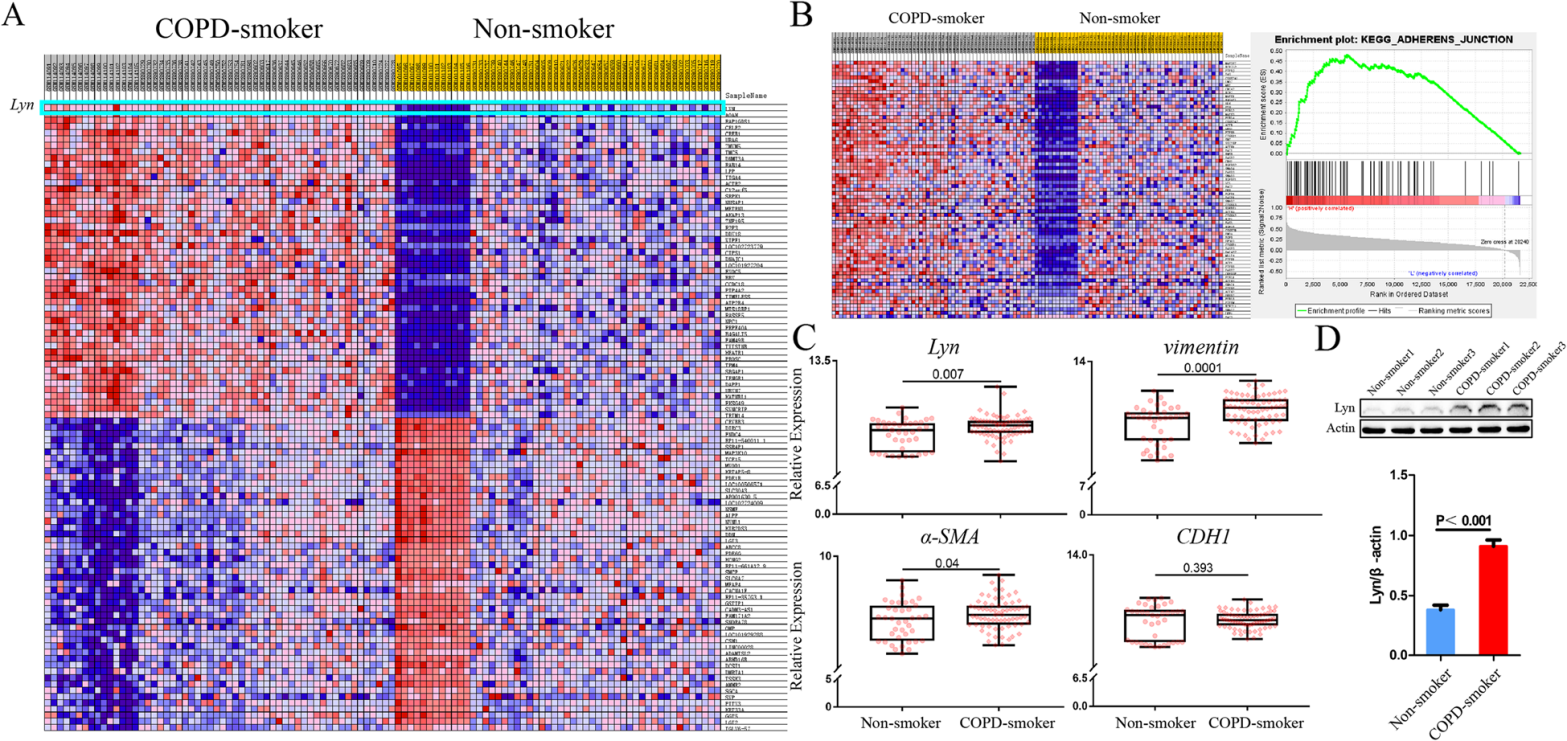
The expression levels of EMT makers correlate with Lyn in COPD-smoker patients.**a** Heat map of differentially expressed genes between COPD-smoker group and Non-smoker group; **b** Single gene GSEA of Lyn, high expression of Lyn was associated with adherens junction; **c** Relative expression of *Lyn*, *vimentin*, *α-SMA* and *CDH1*in Non-smoker and COPD-smoker group; **d** Protein level of Lyn in in Non-smoker and COPD-smoker group

We combined two GEO datasets (GSE108134 and GSE5058) to identify the key genes involved in COPD. Gene Set Enrichment Analysis (GSEA) showed that Lyn kinase expressed differentially between Non-smoker group (*n* = 39) and COPD-smoker group (*n* = 69) (Fig. 1a). Notably, single-gene analysis of Lyn revealed that high expression of Lyn was associated with adherens junction (Fig. 1b). To investigate the correlation between Lyn and EMT in COPD-smoker patients, we analyzed the expression levels of Lyn, vimentin and α-SMA. We found that the expression level of Lyn was significantly up-regulated in COPD-smoker patients compared with Non-smoker (*p* = 0.007, Fig. 1c). Meanwhile, the expression levels of vimentin (*p* = 0.0001) and α-SMA (*p* = 0.04), two molecular markers of EMT, were increased as well. However, there was no difference in the expression of E-cadherin (*P* = 0.393). To validate the microarray data, western blot was applied. It also showed that the protein level of Lyn was significantly increased in COPD-smoker patients (Fig. 1d).

### Cigarette smoke extracts (CSE) induces EMT in a time- and dose-dependent manner

**Fig. 2:**
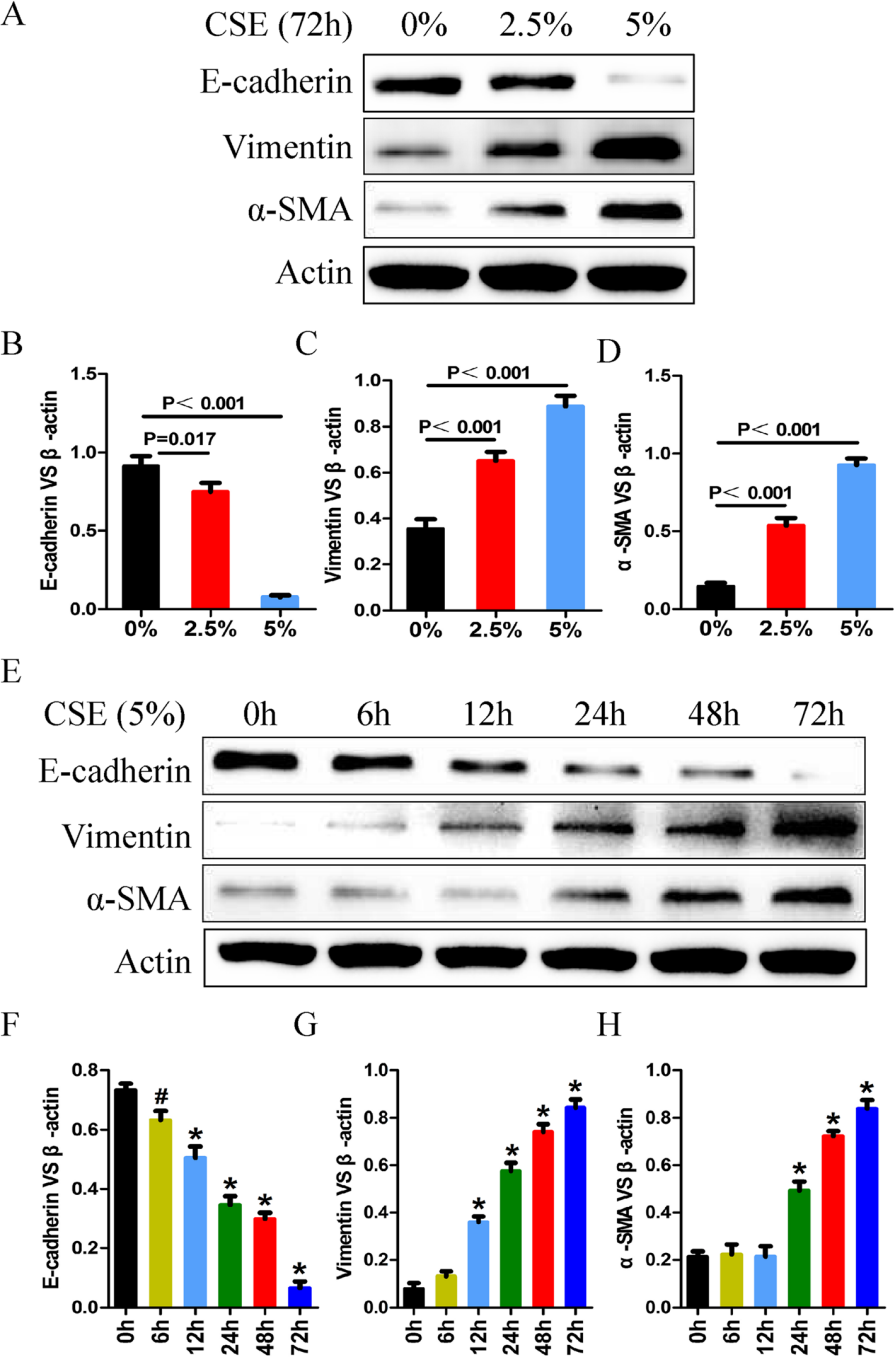
CSE induces EMT in a time- and dose-dependent manner. **a** 16HBE cells were treated with 0, 2.5 and 5% CSE, respectively. Then the expression of EMT markers were detected by western blot; **b**, **c** and **d** Statistic data of each EMT markers in Western blot analysis; **e** 16HBE cells were treated with 5% CSE at different time points; **f**, **g** and **h** Statistic data of each EMT markers in Western blot analysis. The data represent means±s.d. All data are representative of three experiments

Cigarette smoking is one of the most commonly identifiable risk factors for COPD. In order to investigate whether CSE induces EMT in vitro, different time-points or concentrations of CSE were performed in 16HBE cells and expression levels of EMT markers were assessed by using western blot analysis. The data showed that CSE down-regulated E-cadherin protein level and up-regulated vimentin and α-SMA protein levels in a time- and dose-dependent manner (Fig. 2).

### EMT induced by CSE is regulated by activated Lyn

**Fig. 3:**
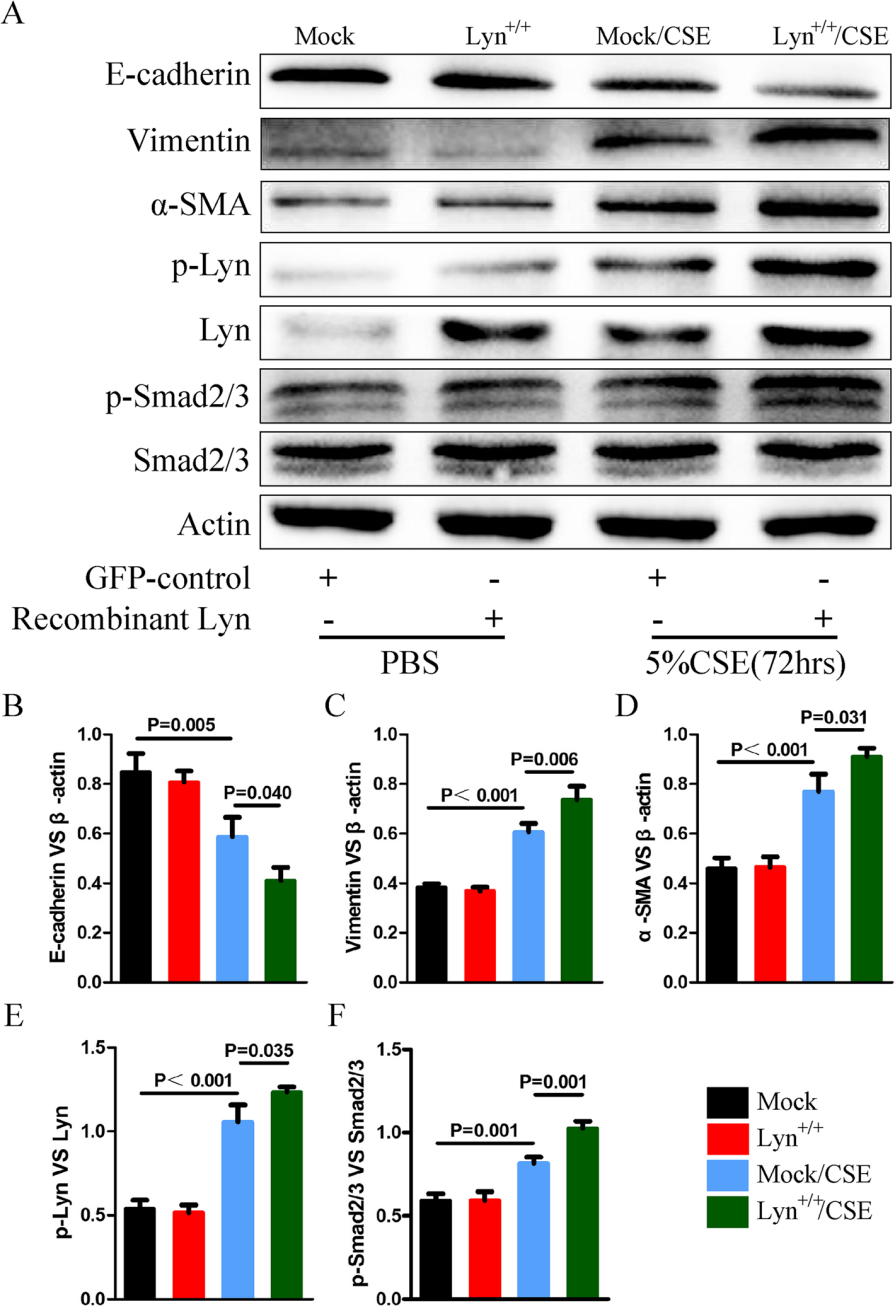
Lyn kinase activity regulates EMT. **a** The expression of EMT markers, Lyn, Smad2/3, and phosphorylation of Lyn and Smad2/3 in 16HBE cells treated with 5% CSE for 72 h were detected by western blot; **b c**, **d**, **e**, and **f** Statistic data of each indicated genes in Western blot analysis. Mock, 16HBE cells were infected with GFP-only virus control. Lyn^+/+^, 16HBE cells were infected with recombinant lentivirus containing Lyn. Mock/CSE, 16HBE cells were treated with 5% CSE after infected with GFP-only virus control. Lyn^+/+^/CSE, 16HBE cells were treated with 5% CSE after infected with recombinant lentivirus containing Lyn. The data represent means±s.d. All data are representative of three experiments

To further explore the role of Lyn in EMT that induced by CSE, Lyn was over-expressed in 16HBE cells and the expression levels of EMT makers were examined by western blot. Cells were treated with 5% CSE for 72 h. Compared with those in mock group, the protein levels of vimentin and α-SMA in mock/CSE and Lyn^+/+^/CSE were increased (Fig. 3a, c and d). In the meantime, E-cadherin protein levels were decreased in mock/CSE and Lyn^+/+^/CSE compared with those in mock treatment (Fig. 3a, b). However, no changes in the protein levels of the three EMT markers were observed between Lyn^+/+^ and mock group (Fig. 3). Therefore, it forced us to detect the phosphorylation levels of Lyn in different groups. It showed that although the protein level of Lyn was up-regulated in Lyn^+/+^ cells, the phosphorylation level of Lyn in this group was not changed (Fig. 3a, e). Whereas the phosphorylation levels of Lyn were increased in mock/CSE and Lyn^+/+^/CSE compared with non-treated groups including mock and Lyn^+/+^ cells (Fig. 3a, e). These results revealed that both the expression and phosphorylation of Lyn were induced by CSE, and the phosphorylation level of Lyn may play a key role in regulation of EMT. Additionally, we found that the phosphorylation levels of Smad2/3 were increased in mock/CSE and Lyn^+/+^/CSE, but there was no difference in Lyn^+/+^ cells (Fig. 3a, f).

### Lyn regulates CSE-induced EMT by phosphorylation of Smad2/3

**Fig. 4:**
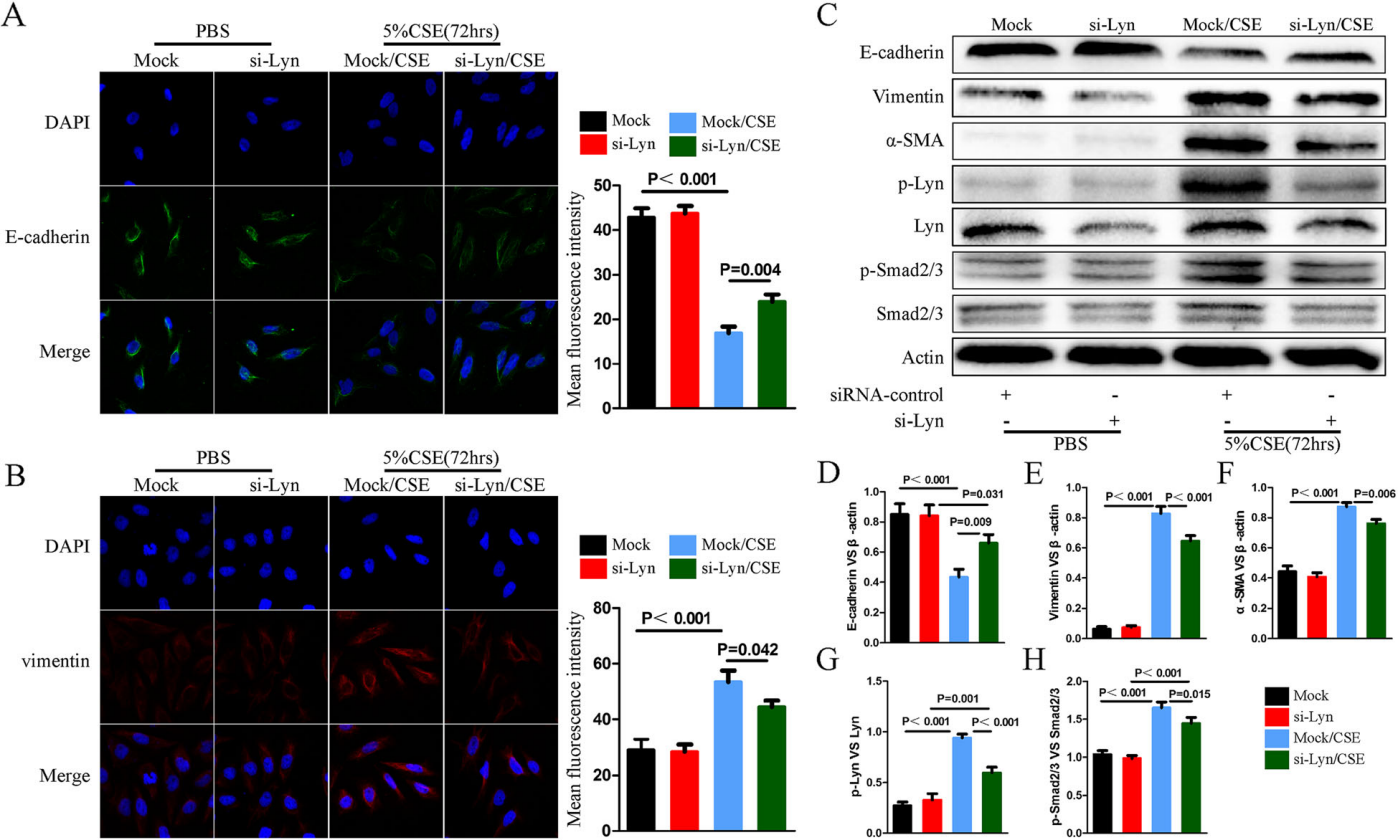
Lyn regulates EMT by phosphorylation of Smad2/3. **a** Immunofluorescence imaging with E-cadherin in green of 16HBE cells in different treatments; **b** Immunofluorescence imaging with vimentin in red of 16HBE cells in different treatments; **c** Western blot analysis of indicated genes; **d**, **e**, **f**, **g** and **h**. Statistic data of each indicated genes in Western blot analysis. Mock, 16HBE cells were transfected with control siRNA. si-Lyn, 16HBE cells were transfected with Lyn siRNA. Mock/CSE, 16HBE cells were treated with 5% CSE for 72 h after transfected with control siRNA. si-Lyn/CSE, 16HBE cells were treated with 5% CSE for 72 h after transfected with Lyn siRNA. The data represent means±s.d. All data are representative of three experiments

Lyn is a kinase, which functions primarily by phosphorylation of downstream effectors. To investigate whether the kinase activity of Lyn is required for the regulation of EMT, the expression of Lyn was inhibited by using siRNA. The EMT markers expression levels were checked by immunofluorescence (Fig. 4a, b). Compared with that in mock/CSE, the fluorescence intensity of E-cadherin in si-Lyn/CSE was increased (*p* = 0.004), whereas the fluorescence intensity of vimentin in si-Lyn/CSE was decreased (*p* = 0.042). Similar results were obtained in western blot analysis (Fig. 4c). The protein level of α-SMA was decreased in si-Lyn /CSE compared with mock/CSE as well (Fig. 4c, f). Furthermore, the phosphorylation level of Lyn in si-Lyn /CSE was significantly down-regulated compared with mock/CSE (*p* < 0.001, Fig. 4c, h). Moreover, the phosphorylation level of Smad2/3, which was increased by CSE, was decreased after the expression and phosphorylation of Lyn were inhibited (*p* = 0.015, Fig. 4c, h). Recent studies discovered that phospho-activated Smad2/3 functions as major transcription factor to mediate EMT [14, 28]. Taken together, these data support that Lyn expression and activity regulate EMT by phosphorylation of Smad2/3.

### Effect of Lyn on EMT of airway epithelial cells in CS-exposed mice

**Fig. 5:**
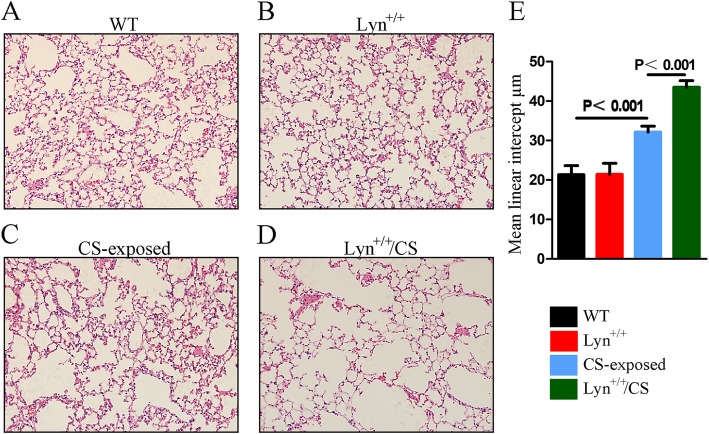
Lyn enhances pathological changes of lung in CS-exposed mice.**a** Wild type mice; **b** Lyn over-expression transgenic mice; **c** CS-exposed mice model in WT background; **d** CS-exposed mice model in Lyn^+/+^ background. **e** Regional emphysema severity was quantified in each sample using the mean linear intercept. WT, wild type mice; Lyn^+/+^, Lyn over-expression transgenic mice; CS-exposed, CS-exposed mice model in WT background; Lyn^+/+^/CS, CS-exposed mice model in Lyn^+/+^ background. All data are representative of three experiments

To confirm that Lyn played a key role in regulation of EMT in CS-induced COPD, Lyn^+/+^ transgenic mice model were constructed and lung injuries were checked. In control and Lyn^+/+^ transgenic mice, no obvious lesions were observed in the lung, and alveolar walls were intact (Fig. 5a, b). But in CS-exposed and Lyn^+/+^/CS groups, the lung had severe lesions (Fig. 5c, d). Additionally, especially in Lyn^+/+^ /CS group, the alveolar walls were thin, the alveoli were expanded (Fig. 5d). In addition, the severity of regional emphysema was measured by mean linear intercept (MLI), Lyn^+/+^ mice exposed to CS had more severe emphysema than any other groups (Fig. 5e). These results showed that typical pathological changes occurred after long-term exposure to CS in mice, especially in Lyn^+/+^ transgenic mice.

**Fig. 6:**
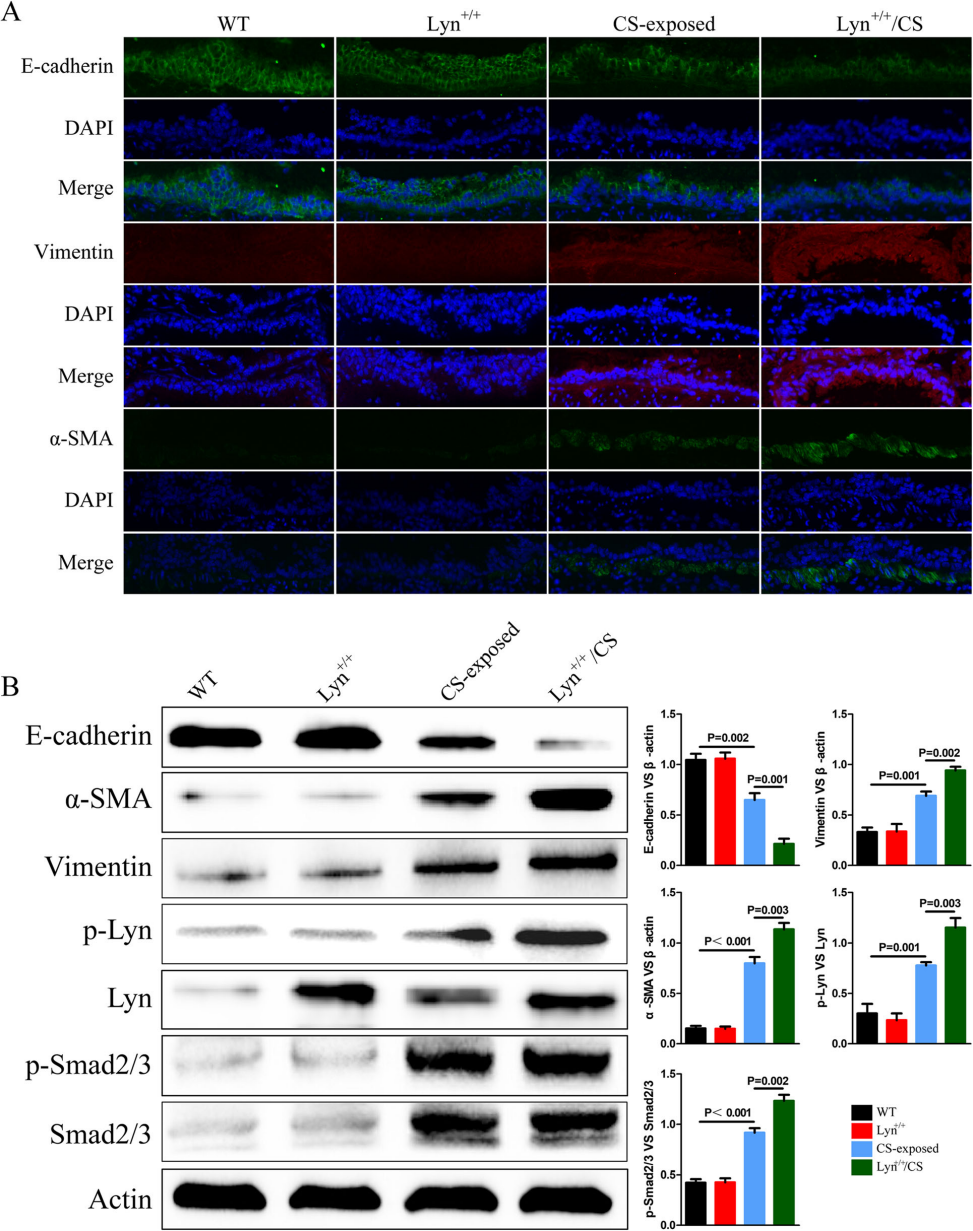
Effect of Lyn on EMT of airway epithelial cells in CS-exposed mice.**a** Immunofluorescence imaging with EMT markers of lung sections; **b** The expression of EMT markers, Lyn, Smad2/3, and phosphorylation of Lyn and Smad2/3 in lung tissue were detected by western blot. WT, wild type mice; Lyn^+/+^, Lyn over-expression transgenic mice; CS-exposed, CS-exposed mice model in WT background; Lyn^+/+^/CS, CS-exposed mice model in Lyn^+/+^ background. The data represent means±s.d. All data are representative of three experiments

In the meantime, the expression levels of EMT makers in airway epithelial cells were assessed by using immunofluorescence. Compared with those in control mice, the fluorescence intensities of vimentin and α-SMA in CS-exposed and Lyn^+/+^ /CS mice were up-regulated, whereas the fluorescence intensities of E-cadherin were down-regulated in CS-exposed and Lyn^+/+^ /CS mice compared with those in control mice (Fig. 6a). Furthermore, western blot was performed to detect the protein levels of EMT markers(Fig. 6b). The protein levels of vimentin and α-SMA were increased in CS-exposed and Lyn^+/+^/CS mice compared with control mice, with the decreased protein levels of E-cadherin. It also showed that the phosphorylation levels of Lyn and Smad2/3 were increased in CS-exposed and Lyn^+/+^/CS mice compared with non-treated groups including control and Lyn^+/+^ mice.

## Discussion

COPD is in the spotlight for the high prevalence and morbidity, and the high burden of COPD associated with cigarette smoking has been increasing worldwide [29]. A study of systematic review and meta-analysis showed that the prevalence rate of COPD was obviously higher in smokers compared with that in non-smokers during 1990–2004 across 28 countries, and COPD affected up to 50% of all long-term smokers [30–32]. It is believed that cigarette smoking contributes to structural changes in the airways during COPD progression and initiates airway remodeling, a well-recognized pathological feature of COPD [4, 33]. Indeed, in our present study, pathological changes and obvious lesions were observed in the lung in CS-exposed mice compared with those in WT mice. Furthermore, in CS-induced groups, alveolar walls were thin, alveoli were expanded, more severe emphysema was observed. It confirmed that cigarette smoking is one of the main risk factors for COPD.

EMT is involved in changes that lead to loss of cell–cell adhesion and cell polarity, with acquisition of migratory and invasive properties, which occurs during wound healing, tissue regeneration, organ fibrosis and tumor progression. Now EMT is thought to be involved in COPD pathology, which could contribute to small airway narrowing and airflow obstruction [34, 35]. The gain of mesenchymal markers like vimentin and α-SMA with a concomitant loss of E-cadherin are hallmarks of EMT. Recent studies showed that cigarette smoking induced EMT in alveolar type II cell line A549 and bronchial epithelial cell line BEAS2B [36, 37]. In our present study, in order to estimate the effect of CSE on EMT, 16HBE cells were treated with CSE at different time-points or concentrations. The result showed that CSE induced EMT in 16HBE cells in a time- and dose-dependent manner. Meanwhile, in vivo study also confirmed that EMT was able to be induced in CS-induced groups. Therefore, EMT might be a new target in anti-COPD drug discovery.

Src family kinases (SFKs) are the largest family of non-receptor tyrosine kinases, which have a major role in cellular events of different biological systems, such as immune system and nervous system [38, 39]. Besides the fundamental roles in cell proliferation and survival, SFKs enhance cell migration and invasion across different cancers through EMT [40]. Lyn is one of the SFK members, functions as molecular switches that control immune receptors to direct homeostasis or inflammation. Studies have shown that there are correlations between increased expression of Lyn and mesenchymal phenotype in cancer cells [41, 42]. However, a correlation between expression of Lyn and EMT in CE-induced COPD has not been defined well. By analyzing GEO datasets, we found the expression level of Lyn was up-regulated, and the expression of EMT markers like vimentin and α-SMA were increased as well. Although the expression level of E-cadherin was not changed in this GEO data, previous studies have revealed that its expression was decreased in COPD patients and we also validated it in the cell and animal models [6, 7]. Furthermore, the increasing range of vimentin and α-SMA, and the decreasing range of E-cadherin were enhanced in Lyn^+/+^/CS mice and in Lyn^+/+^ cells treated with CSE. In the meantime, after knockdown Lyn using siRNA, the expression levels of EMT markers were recovered in CSE treatment. According to these data, we believed that the expression of Lyn correlates with the expression of E-cadherin, vimentin and α-SMA in CS-induced COPD.

Like other Src family members, Lyn has a kinase domain along with two protein interaction domains. Lyn regulates various cellular processes by phosphorylation of inhibitory receptors, enzymes, and adaptors through its kinase activity [43, 44]. In our study, we discovered not only the expression of Lyn was increased in CS-exposed model, but also the phosphorylation of Lyn was up-regulated. However, no changes in phosphorylation of Lyn were observed in non-treatment group, neither in Lyn^+/+^ mice nor in Lyn^+/+^ cells. This leads us to convince that the phosphorylation level of Lyn plays a key role in promoting EMT in CS-exposed model. Importantly, the phosphorylation of Smad2/3 was enhanced by activated Lyn in Lyn^+/+^/CS mice and in Lyn^+/+^ cells treated with CSE, and the phosphorylation of Smad2/3 was decreased after Lyn knockdown in CSE treatment. On one hand, we speculated that Lyn was able to phosphorylate Smad2/3 directly through its kinase activity, and there might be protein-protein interactions between Lyn and Smad2/3. On the other hand, Lyn phosphorylated Smad2/3 in an indirect manner, in which Lyn functioned as a mediator to regulate the increasing phosphorylation of Smad2/3. But both of the hypotheses need to be further validated. In addition, transforming growth factor (TGF)-β, a multifunctional cytokine, has been implicated as a driver of COPD airway pathology [45, 46]. It is widely known that TGF-β activates the phosphorylation of Smad2/3 by binding to its receptors. Then the phosphorylated Smad2/3 enters into nucleus with Smad4, and regulates the expression levels of downstream genes. Therefore, there might be a potential relationship between TGF-β and Lyn. The possible role of TGF-β in regulation of Lyn or the function of Lyn in TGF-β signaling pathway still needs to be investigated.

**Fig. 7:**
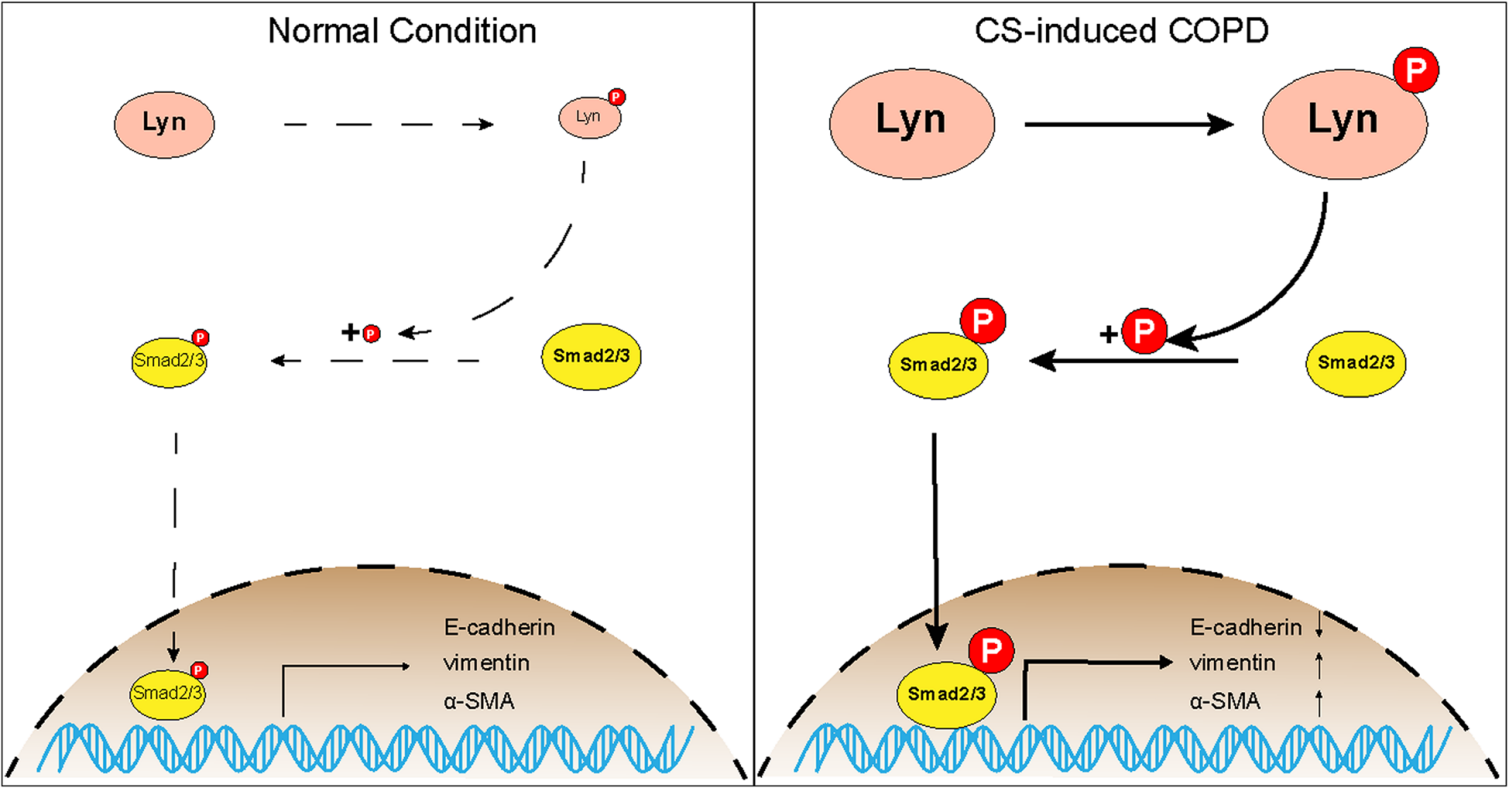
Schematic of Lyn regulates EMT through Smad2/3 signaling in CS-exposed model. In CS-exposed model, not only the expression of Lyn, but also the phosphorylation of Lyn was increased. Then phosphorylation of Smad2/3 was up-regulated by activated Lyn through a direct or indirect way, and more phosphorylated Smad2/3 moved into nucleus. In nucleus, phosphorylated Smad2/3 functioned as major transcription factors to regulate the expression of EMT makers, promoted EMT in CS-exposed model

In summary, in CS-exposed model, not only the expression of Lyn, but also the phosphorylation of Lyn was increased. Then phosphorylation of Smad2/3 was up-regulated by activated Lyn through a direct or indirect way, and more phosphorylated Smad2/3 moved into nucleus. In nucleus, phosphorylated Smad2/3 functioned as major transcription factors to regulate the expression of EMT makers, promoted EMT in CS-exposed model (Fig. 7). Current methods for treating COPD are long-term drug maintenance with inhaled corticosteroid and bronchodilators. In this study, we found that EMT in COPD is regulated by activated Lyn through phosphorylation of Smad2/3. Therefore, specific targeting of Lyn might be a potential therapeutic approach to treat COPD by inhibition of EMT.

## Conclusion

By analyzing GEO data, we found that high expression of Lyn was associated with adherens junction. Lyn expression was up-regulated in COPD-smoker patients, which was validated by western blot. In addition, the gain of vimentin and α-SMA with a concomitant loss of E-cadherin correlated with Lyn expression in both cell and mouse models. Importantly, we found that Lyn regulated EMT by phosphorylation of Smad2/3. As a kinase, Lyn is “druggable”. Therefore, our research might provide a new method to treat COPD by targeting Lyn kinase specifically.

## Data Availability

The datasets generated during and/or analyzed during the current study are available from the corresponding author on reasonable request.

## Abbreviations

COPD: Chronic obstructive pulmonary disease
CSE: Cigarette smoking extracts
EMT: Epithelial–mesenchymal transition
GEO: Gene expression omnibus
H&E: Hematoxylin-eosin staining
LPS: Lipopolysaccharide
SFKs: Src family kinases
WT: Wild type
